# Genetic and phenotypic differentiation of an Andean intermediate altitude population

**DOI:** 10.14814/phy2.12376

**Published:** 2015-05-06

**Authors:** Christina A Eichstaedt, Tiago Antão, Alexia Cardona, Luca Pagani, Toomas Kivisild, Maru Mormina

**Affiliations:** 1 Division of Biological Anthropology, University of CambridgeCambridge, Cambridgeshire, UK; 2 Centre for Pulmonary Hypertension, Thoraxclinic at the University Hospital HeidelbergHeidelberg, Baden-Württemberg, Germany; 3 Department of Vector Biology, Liverpool School of Tropical MedicineLiverpool, Lancashire, UK; 4 Wellcome Trust Sanger InstituteHinxton, Cambridgeshire, UK; 5 Faculty of Humanities and Social Sciences, University of WinchesterWinchester, Hampshire, UK; 6 School of Chemistry, University of East AngliaNorwich, Norfolk, UK

**Keywords:** Calchaquíes, Diaguita, lung capacity, moderate hypoxia

## Abstract

Highland populations living permanently under hypobaric hypoxia have been subject of extensive research because of the relevance of their physiological adaptations for the understanding of human health and disease. In this context, what is considered high altitude is a matter of interpretation and while the adaptive processes at high altitude (above 3000 m) are well documented, the effects of moderate altitude (below 3000 m) on the phenotype are less well established. In this study, we compare physiological and anthropometric characteristics as well as genetic variations in two Andean populations: the Calchaquíes (2300 m) and neighboring Collas (3500 m). We compare their phenotype and genotype to the sea-level Wichí population. We measured physiological (heart rate, oxygen saturation, respiration rate, and lung function) as well as anthropometric traits (height, sitting height, weight, forearm, and tibia length). We conducted genome-wide genotyping on a subset of the sample (*n* = 74) and performed various scans for positive selection. At the phenotypic level (*n* = 179), increased lung capacity stood out in both Andean groups, whereas a growth reduction in distal limbs was only observed at high altitude. At the genome level, Calchaquíes revealed strong signals around *PRKG1*, suggesting that the nitric oxide pathway may be a target of selection. *PRKG1* was highlighted by one of four selection tests among the top five genes using the population branch statistic. Selection tests results of Collas were reported previously. Overall, our study shows that some phenotypic and genetic differentiation occurs at intermediate altitude in response to moderate lifelong selection pressures.

## Introduction

At high altitude (HA) hypobaric hypoxia causes a decrease in the partial pressure of inspired oxygen and leads to low availability of oxygen in the body, which reduces fitness and can affect survival (Frisancho 1975). However, other stressors such as increased radiation, aridity, broader temperature ranges, and poorer diets may also affect developmental characteristics in these environments (Leonard 1989; West et al. 2007; Pomeroy et al. 2014). Evidence for HA adaptation was shown in Tibetans (Beall et al. 2010; Bigham et al. 2010; Yi et al. 2010; Peng et al. 2011; Simonson et al. 2012), Andeans (Beall et al. 2001; Bigham et al. 2009, 2010; Zhou et al. 2013; Eichstaedt et al. 2014), and Ethiopians (Alkorta-Aranburu et al. 2012; Scheinfeldt et al. 2012; Huerta-Sanchez et al. 2013; Udpa et al. 2014), thus demonstrating the effects of long-term exposure to hypoxia above 3000 m. However, little is known about the effects of moderate levels of hypoxia on human physiology, such as those found at intermediate altitudes (IA), which we define hereafter as altitudes between 2000 and 3000 m. Some indication that moderate hypoxia may have a physiological impact comes from evidence of an albeit modest occurrence of Acute Mountain Sickness (AMS), High Altitude Pulmonary Edema (HAPE) (West et al. 2007), and birth weight reduction (Mortola et al. 2000) just above 2000 m.

There is deep academic interest in the physiology of HA due to its obvious medical relevance; yet, the understanding of the cellular and molecular mechanisms that underlie the physiological response to hypobaric hypoxia remains patchy. We also need to better understand the interplay between hypobaric hypoxia and other co-occurring environmental and socioeconomic stressors, and between phenotypic plasticity and genetic adaptation (Storz et al. 2010). The latter was suggested by a number of studies (Alkorta-Aranburu et al. 2012; Simonson et al. 2012; Bigham et al. 2013; Eichstaedt et al. 2014), and even reported to occur at IA (Pagani et al. 2012; Huerta-Sanchez et al. 2013). However, the functional relevance of these genomic signatures remains largely unassessed.

Given these knowledge gaps, we recently investigated patterns of adaptation to HA in the Collas of Northwest Argentina who live above 3500 m (Eichstaedt et al. 2014). Here, we present phenotypic and genetic data from an intermediate altitude (IA) Andean population at 2300 m, the Calchaquíes. These people inhabit the Calchaquí Valleys, a region bordering the high plateau of the Central Andes in Argentina and characterized by a mild and dry climate (average air temperature in coldest month: 7°C, warmest month: 21°C) (Bianchi and Cravero 2010). This gives us the opportunity to assess the impact of moderate levels of hypoxia upon the organism largely without the confounding effects of other environmental stressors.

Remarkably, there are major adaptive differences between Andeans, Tibetans, and Ethiopians at the phenotypic level. For example, the typical physiological response to hypoxia in Andean highlanders is an increased hematocrit, extended lung volume, blunted hypoxic ventilatory response, and increased pulmonary diffusion (Rupert and Hochachka 2001). At the morphological level, Andeans also show a larger chest and greater lung volume (Brutsaert et al. 1999). At the same time height is reduced, thus increasing the ratio of ventilatory capacity to body mass (Guyton and Hall 2006). Fetal growth restriction in Andeans and Tibetans is limited in comparison to nonnative highlanders born at HA (Giussani et al. 2001; Moore et al. 2011). At the cellular level, the vasodilator nitric oxide (NO) in exhaled breath is 28% elevated in Andeans compared to sea-level values (Beall 2001). Tibetans show even higher values of exhaled NO (Beall 2001; Erzurum et al. 2007), higher alveolar ventilation (Zhuang et al. 1993), no hypoxia induced pulmonary vascular resistance response (Groves et al. 1993) and only minimally increased hemoglobin concentrations (Beall 2006). Ethiopians, on the other hand, display a physiology barely distinguishable from lowlanders with only slightly increased hematocrit (Scheinfeldt et al. 2012), elevated pulmonary arterial pressure and permanently high pulmonary blood flow (Hoit et al. 2011). These different adaptive solutions to hypoxia, suggest convergent evolutionary pathways (Scheinfeldt et al. 2012) and underline the critical importance of hypoxia as an environmental pressure.

Despite this extensive literature, most of the knowledge comes from the study of HA populations, thus it remains unclear whether the same traits have developed under moderate hypoxia. Therefore, our focus on the Calchaquíes allows us to investigate for the first time phenotypic and genetic adaptations in an Andean population at IA, although this is not without challenges. For a start, Calchaquíes are thought to be closely related to other HA populations (Frank 2008), yet their demographic history is poorly researched. The Calchaquíes belong to the Diaguita group of small tribes scattered predominantly in the valleys of Northwest Argentina (1680–3015 m) (De Hoyos 2009). Archeological evidence around the Calchaquí valleys suggest that settlement dates back to 7200 (Rodríguez et al. 2003) and 6500 years ago (Somonte and Baied 2013). Moreover, the region was a migration corridor during the Inca expansion in the XV century; yet, the extent of gene flow that took place at that time is largely unknown. This dearth of population history can potentially complicate the disentangling of demographic and adaptive signals.

From the current HA literature we identify a need for further data to improve our understanding of (i) what are the threshold levels of hypobaric hypoxia with a significant effect on human physiology and development, (ii) whether the same or different mechanisms are triggered by strong and moderate hypoxia, and (iii) whether moderate hypoxia represents a selective pressure strong enough to leave a genomic signature. In order to address these questions we attempt to assess phenotype-genotype correlations by examining, alongside our genome-wide selection tests, a number of anthropometric, and physiological characteristics. In particular, we highlight one candidate gene of selection involved in the NO pathway, as well as physiological and anatomical patterns that could be part of the biological response to the effects of moderate hypoxia.

## Methods

### Subjects and ethical approval

**Figure 1:**
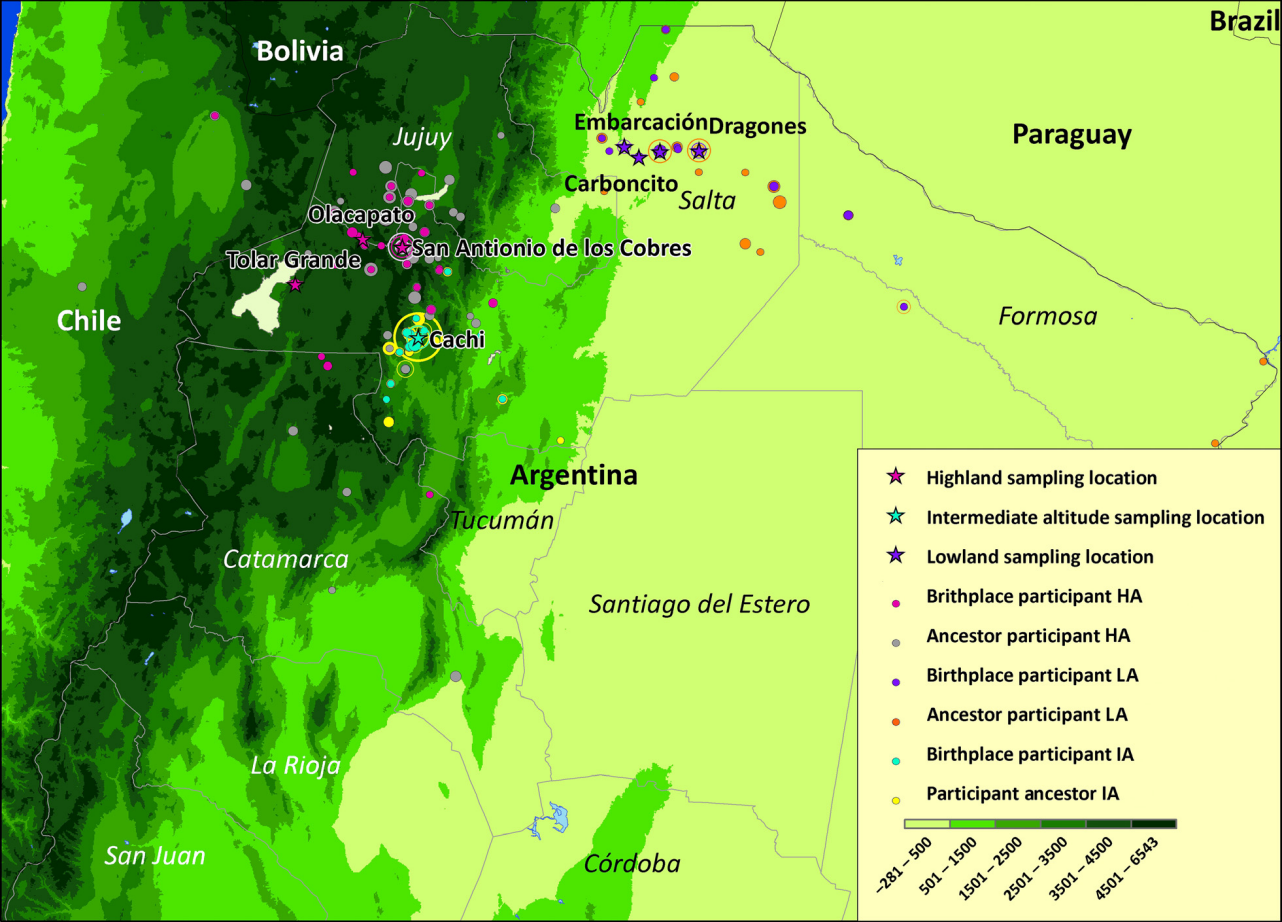
Range of birthplaces of participants and their ancestors in the three regions. Dots on the map are roughly proportional to the number of individuals born in one location. The maximum extent is displayed as circles: 94 Calchaquí participant (IA) ancestors were born in Cachi, 43 Colla participant (HA) ancestors in San Antonio de los Cobres. Stars denote sampling locations. The third purple star from left indicates Misión Chaqueña. HA, high altitude; LA, low altitude; IA, intermediate altitude.

The Calchaquí Valleys lie at intermediate altitude (IA, 2000–3000 m) in the middle of an altitude gradient between the Andean highlands to the West and the Gran Chaco plains to the East (Fig.1). From both of these regions we include a reference population (Collas and Wichí), which has been described elsewhere (Eichstaedt et al. 2010). Our final dataset consisted of 44 Wichí (low altitude, LA, ca. 300 m; 26 females, 18 males), 60 Calchaquíes (IA, 2326 m; 40 females, 20 males), and 75 Collas (HA, >3500 m; 49 females, 26 males), totaling 179 indigenous Argentinean individuals. We only included unrelated adult individuals (>18 years), who were born and raised at the respective altitude and excluded pregnant or lactating women. Ethnicity was self-identified and long-term ancestry was confirmed by identifying birthplaces of participants, their parents, and grandparents. We briefly interviewed each participant to assess general health, age, and the time and type of the last meal. Contents of the meal were assessed in terms of calories with the national nutrient program USDA Food Search (U.S. Department of Agriculture 2008). The total amount of calories of the last meal was added as a variable to the dataset to account for the effects of postprandial hypotension on the physiological measurements taken. Finally, we measured anthropometric and physiological traits and collected saliva samples for genotyping.

The study was approved by the Ethics Committee at the University of East Anglia and the Ministry of Health of the Province of Salta (Ministerio de Salud Pública, Salta, Argentina). All individuals gave written informed consent before taking part in the study. Additional ethical approval for genotyping of the samples at Cambridge Genomic Services was obtained from University of Cambridge Human Biology Research Ethics Committee (HBREC.2011.01).

### Demographic analyses

Saliva sample collection, DNA extraction, and genotyping conditions have been reported elsewhere (Eichstaedt et al. 2014). We chose a subset of 24 Calchaquí (IA) samples with the highest DNA concentrations for genotyping with the Illumina HumanOmniExpress BeadChip for 730,000 Single Nucleotide Polymorphisms (SNPs) for comparison with the previously reported Colla and Wichí samples genotyped using the same platform (Eichstaedt et al. 2014). One lowland sample was excluded due to 50% European admixture and seven samples due to unreported consanguinity. This resulted in a total sample of 20 Wichí, 23 Calchaquí, and 23 Collas being included for genomic analyses. The newly generated Calchaquí sequences (IA) were deposited with NCBI GEO under accession numbers (GSM1363137-GSM1363160).

In order to assess ancestry we surveyed uniparental markers, mitochondrial DNA (mtDNA), and Y-chromosome (Y-chr). We sequenced the hypervariable region I of the mtDNA, between positions 15,908 and 16,498, to identify mitochondrial haplogroups using published primers and protocols (Sykes et al. 1995; Hill et al. 2007). For the Y-chr, we used restriction length polymorphisms (RFLP) to assign samples to Y-chr haplogroups. RFLP analysis for the Native American haplogroups Q and C and European haplogroup R1b, J and K (Corach et al. 2010) were carried out as previously described (Eichstaedt et al. 2014).

We used the software PLINK (Purcell et al. 2007) to determine a set of autosomal SNPs shared between our Calchaquí sample and the following populations: Karitiana, Suruí, Pima, and Colombians from the Human Genome Diversity Project (HGDP) (Li et al. 2008), Han Chinese and Utah residents from the HapMap project (International HapMap-Consortium 2003), Aymara and Quechua published by Mao et al. (2007), Wichí and Collas from Eichstaedt et al. (2014), and a subset of Native American populations (Reich et al. 2012). The final dataset for this ancestry analysis consisted of 17,374 shared SNPs in random association (*r*^2^ < 0.1). We cleaned and phased the data as previously described (Eichstaedt et al. 2014). We obtained admixture proportions using the program ADMIXTURE (Alexander et al. 2009). We performed 100 iterations for *K* values 2–10 to analyze each genome for a given number of potential ancestors. The *K* with the smallest cross-validation error was chosen as the best fit for the data (Alexander and Lange 2011). The same set of SNPs was used for Principal Component Analysis (PCA) using the SmartPCA function implemented in the EIGENSOFT package (Patterson et al. 2006) and to generate genome-wide heterozygosity estimates with the program PLINK.

### Phenotypic measurements

We measured anthropometric traits previously reported to show considerable differences in highland populations: height, sitting height, tibia length, weight, forearm length, and hip circumference. These traits were measured following Frisancho (2008) and Cameron (1981). Height and sitting height were recorded with a stadiometer (Leicester Height Measure, Seca, UK) and weight was recorded with a bio-impedance scale (Body Composition Meter BC-520, Tanita, USA). Hip circumference was measured with tape at the maximum extension of the buttocks (Chasmors Ltd., London, UK) and was used to correct for buttocks soft tissue (Bogin and Varela-Silva 2008). The ratio of height to sitting height was used as a proxy for lower extremity length and body proportions (Martin et al. 1988). Relative subischial leg length (RSLL) was calculated as follows: RSLL = (height − sitting height)/height*100; the lower the RSLL is, the shorter are the legs. Knee height was taken as a proxy for tibia length and forearm length was measured between the styloid process of the ulna and the maximum extension of the elbow. BMI was calculated as weight/(height)^2^. Oxygen saturation and heart rate were measured simultaneously with a digital pulse oximeter (model 8500; Nonin Medical Inc., Plymouth, MN). Values were recorded once they remained stable for at least 10 sec. Respiration rate was determined by counting the number of breaths per minute with the participant seated in a comfortable position. Of these variables, height, weight, BMI, heart rate, respiration rate, and oxygen saturation have been reported previously for a subset of Collas and Wichí included here (Eichstaedt et al. 2014).

Lung function was assessed using a portable spirometer (SpiroPro; CareFusion, Hoechberg, Germany) with disposable mouthpieces (Air Safety Ltd., Morecambe, UK), calibrated daily prior to use. Volume and flow measurements were automatically corrected for body temperature, ambient pressure, and water vapor saturation (BTPS). Procedures were performed according to the guidelines of the American Thoracic Society and European Respiratory Society (ATS/ERS). The highest measurement of at least three attempts of vital capacity (VC), forced vital capacity (FVC), the forced expiratory volume (FEV_1_), and the peak expiratory flow (PEF) were recorded. Reference values for each participant were obtained for the respective gender, height, and age (Quanjer et al. 1993). We created a variable to distinguish healthy individuals from those with reported medical conditions or life style conditions potentially affecting lung function. Thus, participants with acute cold or bronchitis (*n *= 14), alcohol problems (*n *= 1), chest pain during breathing (*n *= 10), dyspnea (*n *= 3), as well as heavy smokers (≥30 cigarettes a month, *n *= 13), were considered “lung affected”. Among the Calchaquíes, 11 individuals were affected (18%), 11 Collas (15%), and 19 Wichí (43%). This variable was considered in all statistical assessments of lung performance.

Phenotypic data were analyzed with SPSS Statistics 21., IBM Corp., New York. We used one-way analysis of variance (ANOVA) to compare continuous and categorical phenotypic variables between the three altitude groups. Levene's homogeneity test was used to determine whether variances were equal. For equal variances Hochberg's GT2 post hoc test was considered, whereas unequal variances were corrected using the Games-Howell post hoc test. The influence of a given independent variable (IV) on a continuous dependent variable (DV) was assessed by type I General Linear Model (GLM). Bonferroni correction was applied to account for multiple testing. Pairwise comparisons of the estimated marginal means of the DV were made with means adjusted for other covariates, that is, continuous variables included in the model.

### Tests for positive selection

We used four different tests (integrated Haplotype Score, iHS; Cross Population Extended Haplotype Homozygosity, XP-EHH; Population Branch Statistic, PBS and the Fixation Index, *F*_ST_) to screen for empirical outliers in our data likely driven by natural selection following the methods described previously (Weir and Cockerham 1984; Sabeti et al. 2007; Pickrell et al. 2009; Yi et al. 2010; Eichstaedt et al. 2014). Similar to Pickrell et al. (2009), we used 200 kb windows in iHS and XP-EHH scans binned by SNP numbers to compensate for unequal SNP distribution in the Illumina OmniExpress SNP-chip data. The top 1% windows of iHS in Calchaquíes were only considered if they were not among the top 5% of Wichí iHS windows. *F*_ST_ and PBS tests, which emphasize maximum SNP values, were performed on 100 kb windows using lowland Wichí population from Argentina and Siberian Eskimos, NCBI GEO series number GSE55586, (Cardona et al. 2014) as outgroups. The top 1% windows of all four tests were compared to an a priori candidate gene list (*n* = 213) details specified in Table S2, Eichstaedt et al. (2014) to identify genes closely implicated in hypoxia response. The top 1% was chosen as a conservative cut-off, compared to 5% used in other studies investigating Andean genetic adaptations (Bigham et al. 2009, 2010). The candidate genes were based on gene ontology (GO) terms associated with hypoxia response (“Cellular response to hypoxia” and “Cellular response to ROS”) and REACTOME pathways (“NO stimulates guanylate cyclase”, “Metabolism of angiotensinogen to angiotensins” and “VEGF signaling”). All genes related to these pathways were included in the final candidate gene list. While further pathways are involved in the hypoxia response we only focused on those potentially most relevant to Andean phenotypes such as an increased hematocrit, blood flow regulations, and vascularization, albeit keeping the number of candidate genes to a minimum. This list is not exhaustive and other pathways might also be relevant; however, we decided to limit genes to a minimum due to the paucity of relevant functional genetic variations in Andeans. Allele frequencies of highly distinct alleles highlighted by PBS were investigated in the three Argentinean populations using GLM test in SPSS. Genes in the top 1% windows of iHS, XP-EHH, and PBS were screened for enrichment of functional categories by GO terms using the Expression Analysis Systematic Explorer (EASE) score <0.01. The EASE score is a conservative adjustment of Fisher's exact *P*-value implemented in DAVID and more robust for pointing out enriched biological functions (Hosack et al. 2003).

## Results

### Genetic structure and recent admixture in Andean populations

**Figure 2:**
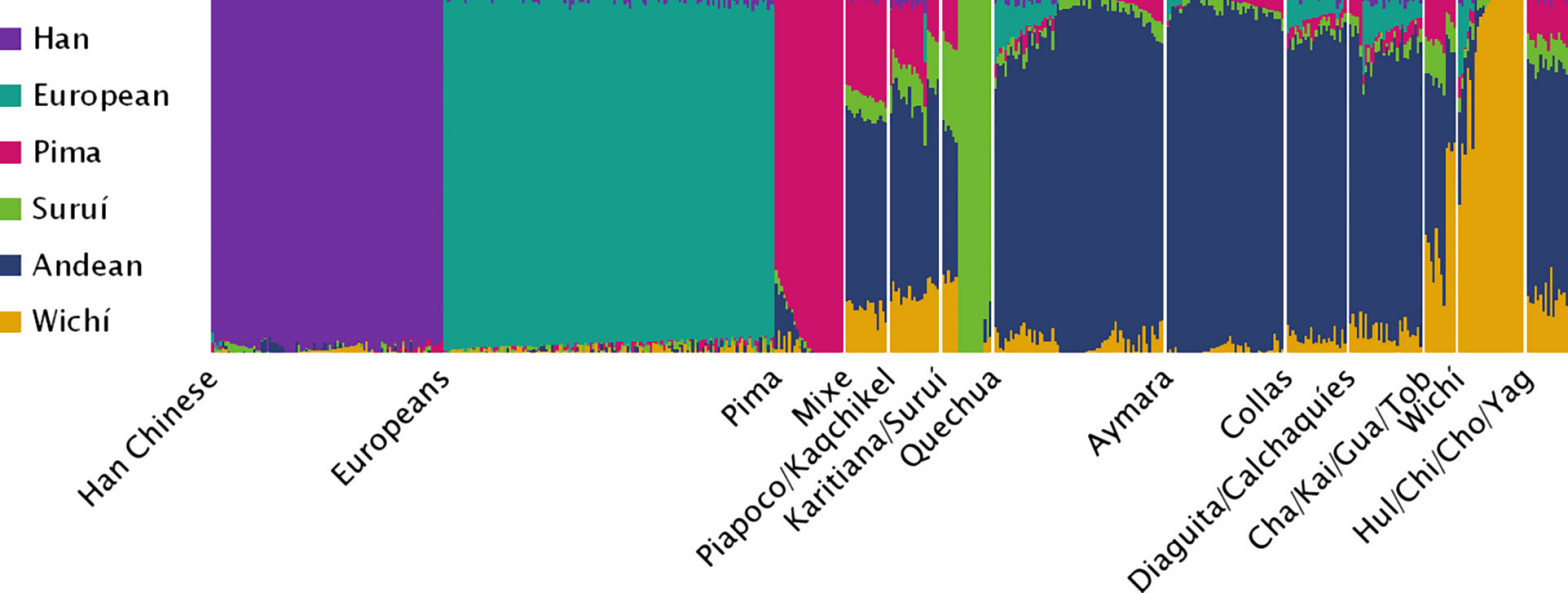
Admixture proportions of Calchaquíes among Native Americans, Han Chinese, and Europeans at *K* = 6. Ancestry components are assigned to each individual. Cross-validation error was the lowest for *K* = 6, which indicates that the population structure observed among the included populations is best explained by 6 distinct ancestry components. Han Chinese, Europeans, Pima, Suruí, Andeans (HA and IA), and Wichí (LA) are characterized by a unique ancestry components; that is, the program assumes a major genetic contribution from separate ancestors for each of these populations. Mixe from Mexico, Piapoco from Colombia, Kaqchikel from Guatemala, and Karitiana from Brazil share the same four Native American admixture components in similar proportions. Chileans (Hul/Chi/Cho/Yag: Hulliche, Chilote, Chono, and Yaghan) display the same four components but the Andean ancestry component accounts for more than 50%. Populations from the Gran Chaco region other than Wichí (Cha/Kai/Gua/Tob: Chané, Kaingang, Guaraní, and Toba) share more than 30% of the Wichí ancestry component.

Places of birth of participants, their parents, and grandparents were used to ascertain long-term residency at each sampling altitude (Fig.1). Admixture analyses were conducted to estimate the extent of recent European gene flow in our sample and to assess genetic differentiation between Calchaquíes (intermediate altitude, IA), other Andean populations, and Native Americans in general (Fig.2). Cross-validation (CV) error and log-likelihood difference indicated *K* = 6 as the best fit for the data. *K* refers to the number of ancestry components or distinct putative ancestors that best explain the observed genomic diversity in the included set of populations. The autosomal European admixture proportion in the Calchaquí population was estimated as 9% on average (Table1). Admixture patterns in Calchaquíes resemble other Andean populations such as Quechua (HA), Aymara (HA), Collas (HA), and Diaguita (IA) (Fig.2). Calchaquíes show the highest heterozygosity of all analyzed Native American populations at 28% (lowest heterozygosity found in Brazilian Suruí with 22% and a mean of 26% across Native American populations, data not shown). This is partly accounted for by the high levels of autosomal European admixture but also indicating lower levels of inbreeding and genetic drift.

**Table 1:** Autosomal admixture estimates at *K* = 6 in Argentineans

Admixture	Wichí (LA), %	Calchaquíes (IA), %	Collas (HA), %
Mean	2.7	8.6	5.0
Median	0.0	8.6	4.9
Range	0.0–20.9	4.1–20.7	0.0–9.5

**Figure 3:**
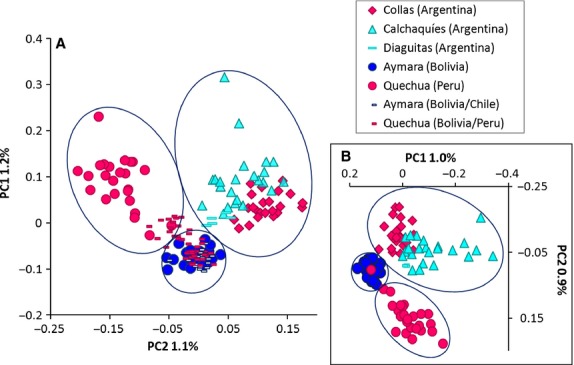
PCA of neighboring Andean populations. Circles denote individuals from Mao et al. (2007); triangles refer to this study, diamonds to Eichstaedt et al. (2014), and rectangles to Reich et al. (2012). (A) Samples overlap but cluster roughly according to geographic origin: populations in the left circle stem from Peru (HA), in the bottom circle from Bolivia and Chile and in the right circle from Argentina (HA and IA). Due to unknown precise sampling locations Quechua individuals sampled by Reich et al. (2012) cannot be assigned to one specific country. However, it is likely that Quechua from Peru cluster with Peruvian Quechua sampled by Mao et al. (2007) and Quechua from Bolivia overlap with Bolivian Aymara. (B) The same samples except for Quechua and Aymara from Reich et al. (2012) with unclear geographic origin were included, showing a clustering by geographic origin apart for one Peruvian Quechua individual.

We performed Principal Component Analysis (PCA) to examine population differentiation. The resulting clustering roughly describes three broad geographic areas (Fig.3): (i) Northwest Argentina including Calchaquíes (this study), highland Collas (Eichstaedt et al. 2014) and Diaguita from an IA (Reich et al. 2012); (ii) Peruvian Quechua (Mao et al. 2007); and (iii) Bolivian and Northern Chilean Aymara (Mao et al. 2007; Reich et al. 2012). The Quechua dataset from Reich et al. (2012) overlapped with the latter two groups because it includes individuals from Bolivia and Peru, suggesting that geography plays a greater role than reported ethnicity in determining the genetic structure of Andean populations.

As autosomal genotype data were only available for a subset of our sample, we analyzed the mitochondrial control region and the Y-chr to investigate haplogroup distribution in the entire dataset and thus obtained complementary ancestry information. All 179 samples clustered within Native American mtDNA haplogroups (Table2). While the majority of Calchaquí Y-chr (80%) belonged to haplogroup Q, which is common Native Americans, a minor subset (20%) of the samples showed affinities to haplogroups that are common in Europe (Table2).

**Table 2:** Mitochondrial DNA and Y-chromosome haplogroups among Argentineans

Marker	Haplogroup	Wichí (LA) (%)	Calchaquíes (IA) (%)	Collas (HA) (%)
Mitochondrial DNA	A	0	0	1 (1.3)
	A2*	11 (25.0)	9 (15.0)	13 (17.3)
	B2	19 (43.2)	34 (56.7)	49 (65.3)
	C1	0	5 (8.3)	4 (5.3)
	D1	14 (31.8)	11 (18.3)	8 (10.7)
	D4h3a	0	1 (1.7)	0
Y-chromosome	Q	16 (88.9)	16 (80.0)	19 (73.1)
	R1b	2 (11.1)	3 (15.0)	6 (23.1)
	J	0	0	1 (3.8)
	E1b1b1b	0	1 (5)	0

* This haplogroup is a paragroup of A2.

### Phenotypic traits characteristic to an intermediate altitude population in the Andes

We assessed the effect of altitude on anthropometric and physiological variables (Table3). Height was not significantly different between groups but weight and BMI were elevated in Wichí. Sitting height as a proxy for torso length showed no difference between groups, whereas tibia length (approximated by knee height) and forearm lengths were similar in Calchaquíes and Wichí, but significantly reduced in Collas. The whole leg length variation was measured as relative subischial leg length (RSLL). Significant differences were only observed between Collas (HA) and Wichí (LA).

**Table 3:** Phenotypic differences between Wichí, Calchaquíes, and Collas

Dependent variable	Wichí (LA, *n* = 44)	Calchaquíes (IA, *n* = 60)	Collas (HA, *n* = 75)	Pairwise comparison*	Model information†
Age (years)	42.1±14.1	45.9±14.4	42.6±13.6	1: *P*=n.s.	Model: *P*=n.s.
				2: *P*=n.s.	ANOVA
				3: *P*=n.s.	
Height (cm)	159.3±8.4	158.0±9.1	157.5±8.1	1: *P*=n.s.	Model: *P*<0.001
				2: *P*=n.s.	adjusted *R*^2^: 64.1%
				3: *P*=n.s.	IVs: Gender, age
Weight (kg)	74.5±15.7	65.8±13.2	65.5±12.5	1: *P*=n.s.	Model: *P*<0.001
				2: *P*=0.008	adjusted *R*^2^: 15.1%
				3: *P*=0.002	IVs: Gender, age
Body mass index (m^2^/kg)	29.4±5.9	26.3±4.2	26.4±4.5	1: *P*=n.s.	Model: *P*=0.002
				2: *P*=0.002	adjusted *R*^2^: 7.2%
				3: *P*=0.003	IVs: Gender, age
Sitting height (cm)	82.0±4.2	81.9±4.3	82.3±4.1	1: *P*=n.s.	Model: *P*<0.001
				2: *P*=n.s.	adjusted *R*^2^: 52.2%
				3: *P*=n.s.	IVs: Gender, age, hip circumference
Knee height (cm)	49.3±3.0	48.4±3.5	46.7±3.3	1: *P*<0.001	Model: *P*<0.001
				2: *P*=n.s.	adjusted *R*^2^: 54.4%
				3: *P*<0.001	IVs: Gender, age, sitting height, hip circumference
Forearm length (cm)	25.6±1.9	25.0±2.2	24.5±1.6	1: *P*=0.028	Model: *P*<0.001
				2: *P*=n.s.	adjusted *R*^2^: 80.3%
				3: *P*<0.001	IVs: Gender, age, height
Relative subischial leg length (%)	48.5±1.6	48.1±1.5	47.7±1.4	1: *P*=n.s.	Model: *P*<0.001
				2: *P*=n.s.	adjusted *R*^2^: 15.8%
				3: *P*=0.014	IVs: Gender, age
Heart rate	74.3±11.0	70.3±9.0	71.2±10.5	1: *P*=n.s.	Model: *P*=n.s.
				2: *P*=n.s.	adjusted *R*^2^: 0.2%
				3: *P*=n.s	IVs: Gender, age
Oxygen saturation (%)	97.1±1.2	92.7±2.4	87.4±3.2	1: *P*<0.001	Model: *P*<0.001
				2: *P*<0.001	adjusted *R*^2^: 72.6%
				3: *P*<0.001	IVs: Gender, age
Respiration rate	17.5±4.6	18.3±3.3	21.1±5.6	1: *P*=0.001	Model: *P*<0.001
				2: *P*=n.s.	ANOVA, unequal variances
				3: *P*=0.001	corrected
Vital capacity (L)	2.6±1.0	2.9±1.0	2.9±1.1	1: *P*=n.s.	Model: *P*<0.001
				2: *P*=0.005	adjusted *R*^2^: 48.5%
				3: *P*=0.015	IVs: Gender, age, height
Forced vital capacity (L)	2.9±1.0	3.2±1.0	3.4±0.9	1: *P*=n.s.	Model: *P*<0.001
				2: *P*=0.022	adjusted *R*^2^: 60.4%
				3: *P*<0.001	IVs: Gender, age, height, lung health
Forced expiratory volume in 1st second (L)	2.6±0.9	2.9±0.9	3.0±0.8	1: *P*=n.s.	Model: *P*<0.001
				2: *P*<0.001	adjusted *R*^2^: 70.2%
				3: *P*<0.001	IVs: Gender, age, height, lung health
Peak expiratory flow (L/min)	4.6±2.0	6.3±2.3	6.1±2.3	1: *P*=n.s.	Model: *P*<0.001
				2: *P*<0.001	adjusted *R*^2^: 57.8%
				3: *P*<0.001	IVs: Gender, age, height, lung health

* Pairwise comparisons: 1: Calchaquíes vs. Collas, 2: Calchaquíes vs. Wichí, 3: Collas vs. Wichí.

† IVs = Independent variables; Levene's test for homogeneity of variances was nonsignificant, apart for heart rate (*P *= 0.034) and respiration rate (*P *= 0.017). Unequal variances were accounted for with the post hoc test Games-Howell in ANOVA. Gender and age had no significant influence on respiration rate. Values are means and standard variation.

Heart rate at rest was not significantly different between populations. Oxygen saturation reflected differences in exposure to hypobaric hypoxia at the respective altitudes (*P* < 0.001, Table3). Calchaquíes and Wichí did not show significant differences in respiration rate while Collas exhibited a significantly elevated rate in comparison to both populations (*P* = 0.001, Table3). Lung function measurements for Calchaquíes were indistinguishable from Collas (Table3). However, both populations differed significantly from Wichí.

### Haplotype homozygosity and allele differentiation patterns specific to Calchaquíes

Three of the 213 predefined candidate genes (*CMA1,* rank 70; *NOS3,* rank 76; *AGAP3*, rank 76) were found in the top 1% of Calchaquí iHS results, which is, however, not significantly higher than the number of genes expected to be highlighted from a list of randomly chosen genes. Similarly, for XP-EHH only two genes (*TPM1,* rank 62; *ITPR1,* rank 100) from the candidate gene list were identified within regions of extended homozygosity and overall no significant enrichment of the hypoxia candidate genes could be observed in the top ranking windows of haplotype homozygosity tests.

In the results of the frequency based tests the pairwise *F*_ST_ of Calchaquíes and Wichí did not highlight any genes from the hypoxia candidate gene list. PBS, however, revealed three hypoxia genes among the top 1% (*PRKG1, CBS,* and *EPO*, see Table4). Again, this is not significantly different from what would be expected by chance (2.1 genes); however, the important NO pathway mediator *PRKG1* ranked 5th highest and thus deserves further consideration given an increased NO in exhaled breath in Andeans (Beall 2001). There were 61 genes highlighted by at least two tests in Calchaquíes, 11 of which were independently highlighted by the three tests iHS, XP-EHH, and PBS (*ACACB, AP1S1, CHKA, FOXN4, MUC12, MUC17, MUC3A, NDUFS8, SERPINE1, TCIRG1, TRIM56*).

**Table 4:** Hypoxia related top 1% PBS genes in Calchaquíes

Rank	Gene*	Name	Function	Hypoxia association	Window PBS _MAX_: Gene PBS_MAX_
5 and 38	*PRKG1*	Protein kinase, cGMP-dependent	Key enzyme in NO pathway, regulates platelet activation and adhesion, smooth muscle contraction, cardiac function	NO stimulates guanylate cyclase	0.836: 0.836; 0.663: also within the gene
48	*CBS*	Cystathionine-beta-synthase	Protects neurons against hypoxic injury; regulates cerebral blood flow	Cellular response to hypoxia	0.631: 0.631
264	*EPO*	Erythropoietin	Regulates erythrocyte production	Cellular response to hypoxia	0.469: 8 kb downstream (No SNP within the gene)

* 213 candidate genes: *ACE, ACE2, ADAM8, AGAP3, AGT, AJUBA, AKR1C3, AKT1, ANGPT4, ANKRD1, ANPEP, ANXA1, APEX1, APOA4, AQP1, ARG1, ARNT, ATP6AP2, ATP7A, BACH1, BAD, BBC3, BMP7, BNIP3, CA9, CAT, CBS, CCNB1, CCS, CD34, CD36, CDK1, CDK2, CITED2, CMA1, CPA3, CREBBP, CRYGD, CST3, CTSD, CTSG, CTSZ, CUL2, DPEP1, DUOX1, DUOX2, E2F1, ECT2, EGLN1, EGLN2, EGLN3, EGR1, ENPEP, EP300, EPAS1, EPO, EPX, ETS1, FABP1, FAM162A, FANCC, FBLN5, FER, FIGF, FLT1, FLT4, FMN2, FNDC1, FOS, FOXO1, FXN, GATA6, GNB1, GNGT1, GPX1, GPX3, GUCY1A2, GUCY1A3, GUCY1B2, GUCY1B3, GZMH, HBA1, HBB, HDAC6, HGF, HIF1A, HIF1AN, HIF3A, HIPK2, HMOX1, HP, ICAM1, IL18, IL18BP, IL6, IRAK1, IREB2, ITPR1, KCNK3, KCNMA1, KCNMB1, KCNMB2, KCNMB3, KCNMB4, KDR, KLF2, LCN2, LMNA, LPO, MAP3K5, MAPK7, MDM2, MDM4, MET, MGARP, MME, MPO, MPV17, MRVI1, MT3, MTOR, MYOCD, NDRG1, NET1, NFE2L2, NKX3-1, NOS1, NOS2, NOS3, NOTCH1, NPEPPS, NRP1, NRP2, PARK7, PAX2, PDE10A, PDE11A, PDE1A, PDE1B, PDE2A, PDE3A, PDE3B, PDE5A, PDE6A, PDE6B, PDE6G, PDE9A, PDGFC, PDIA2, PDK1, PDK2, PDK3, PGF, PKD2, PLEKHA1, PLK3, PMAIP1, PPARGC1B, PPIF, PRDX1, PRDX2, PRDX3, PRDX5, PRDX6, PRKAA1, PRKCE, PRKG1, PRKG2, PTGIS, PTGS2, PTPRK, PXDN, PXDNL, PXN, RBX1, REN, RGCC, RHOB, ROMO1, RPS27A, S100B, SFRP1, SFTPC, SIRT1, SLC29A1, SLC8A1, SOD1, SOD2, SOD3, STC1, STC2, TCEB1, TCEB2, TNFAIP3, TP53, TPM1, TPO, TWIST1, TXNRD1, UBA52, UBB, UBC, UBE2D1, UBE2D2, UBE2D3, UBQLN1, UCN2, UCN3, USP19, VEGFA, VEGFB, VEGFC, VHL*.

### Functional analysis of selected genes

We assessed the genes in the top 1% windows of iHS, XP-EHH, and PBS to identify possible enrichment of functional categories by gene ontology (GO) terms. Among the GO terms, two were related to NO and four were involved in stress response (Table5). Eleven GO terms were involved in synaptic activity, neuronal structure, neuron differentiation or related to the inhibitory neurotransmitter *γ*-aminobutyric acid (GABA) (Table5).

**Table 5:** Subset of enriched GO terms of iHS and XP-EHH results in Calchaquíes

Test (enriched GO terms)	Category	GO term	Enrichment	EASE score
iHS (115)	Nitrogen related	Regulation of nitrogen compound metabolic process	38/1950	0.0055
		Cellular nitrogen compound metabolic process	42/2254	0.0063
	Stress response	Response to biotic stimulus	14/355	0.0008
		Response to DNA damage stimulus	14/379	0.0015
		Defense response	17/534	0.0016
		Response to stress	29/1374	0.0082
	Neuron related	Regulation of neuron projection development	9/168	0.0020
		Regulation of neurogenesis	11/307	0.0078
		Regulation of neuron differentiation	10/260	0.0080
XP-EHH (25)	Neuron related	Synaptic transmission, gabaergic	4/4	0.0001
		Postsynaptic membrane	9/164	0.0009
		GABA-A receptor activity	4/16	0.0013
		GABA signaling pathway	4/17	0.0016
		GABA receptor activity	4/19	0.0021
		Synaptic membrane	9/191	0.0023
		Neuron–neuron synaptic transmission	5/44	0.0024
PBS (27)	Neuron related	Neuron differentiation	2.4	0.0053

### Genotype–phenotype associations

Genotype–phenotype associations were calculated for the SNP in the *PRKG1* intron that had the highest PBS score within the 5th ranking window. Even though we did not assess NO levels, cardiovascular measurements such as blood pressure and heart rate could be affected by differential vasodilation. However, we found no significant correlation between these traits and the allele frequency of the *PRKG1* intronic SNP.

### Signatures of selection at intermediate and high altitude

**Figure 4:**
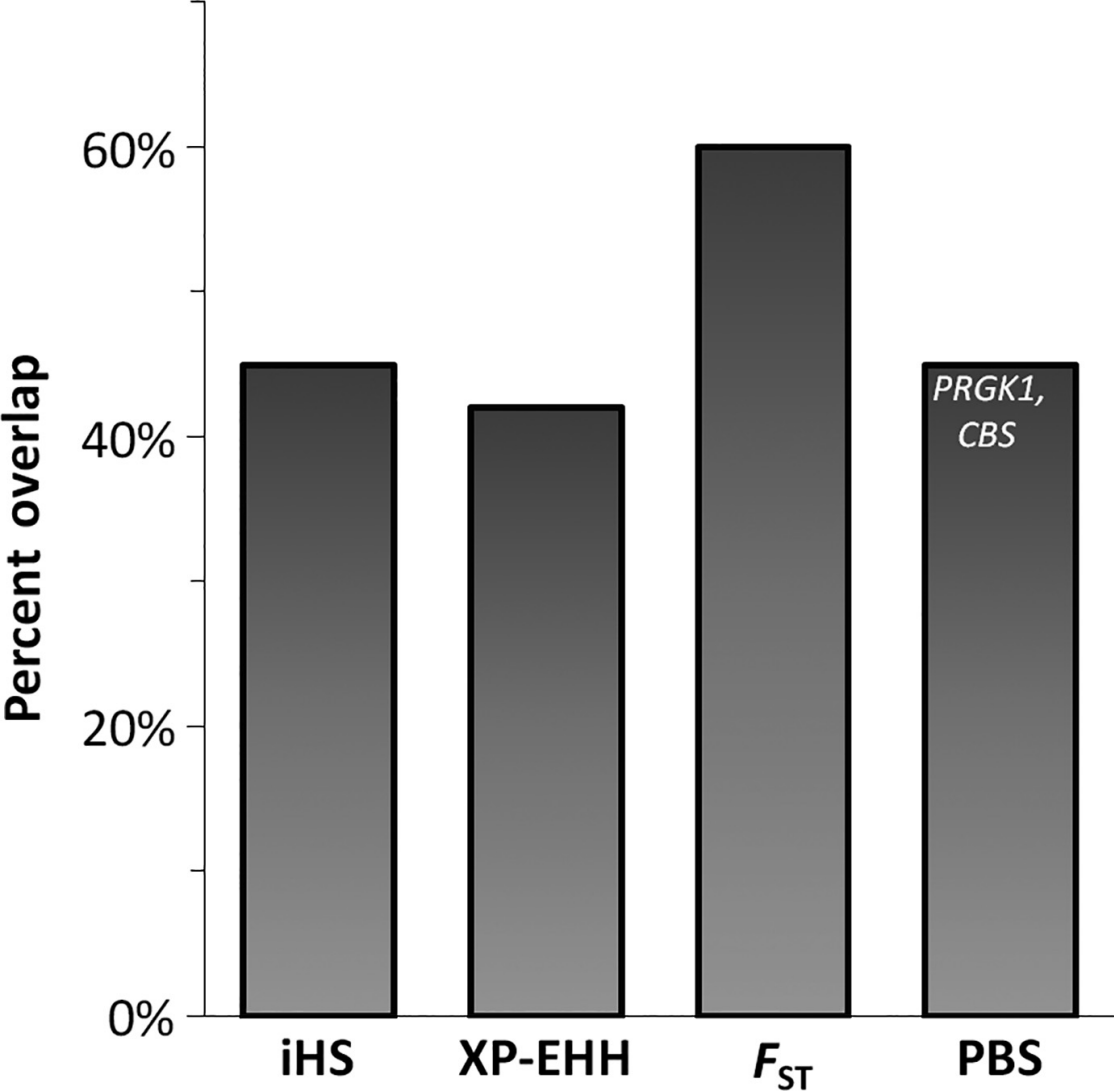
Overlap of the top 1% selection test results in Calchaquíes and with the top 5% in Collas. The overlap of windows highlighted by selection tests was the highest for *F*_ST_ with 60%. However, only two out of 213 candidate genes (*PRKG1* and *CBS*) overlapped, both discovered using PBS. *PRKG1* ranked higher in Calchaquíes with a PBS score of 0.836 (rank 5) than in Collas with a score of 0.693 (rank 52). Thus, allele frequencies were more diverged in Calchaquíes than in Collas. The PBS score is based on the same SNP (rs10740406) for both populations. *CBS* ranked lower in Calchaquíes (rank 48, PBS score 0.632) than in Collas (rank 2, PBS score 1.005), indicating a less pronounced allele differentiation in Calchaquíes in comparison to Collas for this gene. For *CBS* different SNPs within the same 100 kb window were highlighted in the two populations. *CBS*, cystathionine-beta-synthase; *F*_ST_, Fixation Index; iHS, integrated Haplotype Score; PBS, Population Branch Score; *PRKG1*, protein kinase, cGMP-dependent, type I; XP-EHH, Cross Population Extended Haplotype Homozygosity test.

We compared the results of the four selection tests of IA Calchaquíes to our previous findings in the neighboring HA Colla population (Eichstaedt et al. 2014). Overall, the high genome-wide similarity among Collas and Calchaquíes was also reflected in the relatively high sharing of windows in the top 5% (Fig.4). Despite this high window overlap between Calchaquíes (IA) and Collas (HA) only two candidate genes were shared: *PRKG1* and *CBS*.

We found some indication of reduced selection pressure at moderate hypoxia in the genome-wide signatures of Calchaquíes (IA). First, 61 genes were highlighted by more than one test in Calchaquíes (data not shown), in contrast to 108 in Collas (HA). Second, pairwise *F*_ST_ resulted in 17 *F*_ST_ windows in Calchaquíes above a cut-off of *F*_ST_ = 0.6, compared to 40 windows in Collas. Lastly, of the PBS windows overlapping in the top 1% of Calchaquíes and Collas only 22% of Calchaquí branch lengths exceeded those of Collas (*PRKG1* included) indicating a stronger selection pressure in Collas for 78% of commonly highlighted genes. Taken together, these results suggest different strengths of hypoxia-driven selection are acting upon the genome of each population, resulting in weaker signatures at intermediate altitude.

## Discussion

Our aim was to investigate biological adaptation to moderate levels of hypoxia at intermediate altitude (IA) and to characterize the phenotypic and genetic changes that are likely a response to this specific environmental pressure. There is limited evidence of adaptation by natural selection in the genome of Calchaquíes (IA) and support for at least developmental plasticity at the phenotypic level.

Admixture analyses (Fig.2) suggest that all Andean populations included in this study (HA: Quechua, Aymara, Collas; IA: Diaguita, and Calchaquíes) share a highly homogenous genetic makeup, compared to other Native Americans. This is consistent with recently shared ancestry, gene flow or both. However, despite being highly homogeneous, these different Andean groups also display some level of geographic differentiation (Fig.3). While the two Argentinean groups are closer to each other than either is to Peruvian or Bolivian highlanders, a rough distinction between the two populations is still possible (Fig.3).

European admixture in Calchaquíes (IA) was low (Table1) both at the autosomal level (9%) as well as in the mtDNA (none) and Y-chr (20%). In contrast, a study of urban Argentinean populations estimated European admixture to be around 80% in the autosome, 45% in the mtDNA, and even 95% in the Y-chr (Corach et al. 2010).

The effect of moderate hypoxia on the phenotype was assessed using anthropometric and physiological data. Calchaquíes' stature (IA) is comparable to that of Wichí (LA) and Collas (HA). While short stature is expected in the HA group, the reduced growth in the LA sample may be explained by nutrition and disease (Frisancho et al. 1975), and the general poorer health status observed among Wichí. The stature of a group of Turkish children living at similar intermediate altitude was shown to be significantly shorter in comparison to a lowland control group (Malkoc et al. 2012). This might indicate influence of moderate hypoxia on growth patterns while also socioeconomic factors and different growth spurt patterns in children may play a role.

While overall stature was similar in our study, we observed differences in distal limbs length, this being similar between Calchaquíes (IA) and Wichí (LA) but significantly shorter in Collas (HA). While cold has been suggested to reduce the overall length of limb segments (Allen 1877; Katzmarzyk and Leonard 1998), hypoxia likewise exerts a strong effect upon these traits (de Meer et al. 1995; Tripathy and Gupta 2007). The tibia is more susceptible to periods of poorer health than the femur (Bogin and Varela-Silva 2008), thus also reduced oxygen supply is likely to impact bone growth at a critical time during development (Bailey and Hu 2001). Reduced tibia length was also observed among Tibetan and Peruvian children at HA (Stinson and Frisancho 1978; Bailey and Hu 2001; Pomeroy et al. 2012), the latter also showing reduction of forearm length. This suggests hypoxia's effect on distal limb segments, though other climatic factors cannot be excluded. In Calchaquíes (IA), exposure to moderate hypoxia and milder climate (Bianchi and Cravero 2010) may exert a weaker pressure on bone growth than in Collas (HA). Their reduction in distal limb lengths, however, may not be adaptive per se but may indicate the prioritization of oxygen use throughout the organism (Pomeroy et al. 2012), alongside other physiological strategies such as the increased hematocrit, which although was not measured in our sample, is well documented among Andeans (Frisancho 1975; Engström et al. 2010). While tibia and forearm length reduction was only observed in highland Collas, lung volume was increased in Collas and Calchaquíes (IA) alike (Table3). Increased lung capacity is one of the typical Andean adaptive characteristics and it is thought to be a developmental consequence of early exposure to hypoxia (Frisancho 2013), though genetic factors may also be at play as suggested by the significant differences in forced vital capacity (FVC) that exist between native highlanders, acclimatized Europeans, and Europeans raised at HA (Brutsaert et al. 2000).

Shared ancestry between HA and IA Andeans may confound evidence of positive selection. However, despite the extensive genomic similarity (Figs.3), we identified only two shared candidate genes (*CBS* and *PRKG1*). In particular, *PRKG1* is our strongest candidate of selection at IA; however, we could not correlate this gene with any of the phenotypic traits observed. This may be due to our decision not to measure NO-induced vasodilation due to feasibility concerns during the research design.

We also found little overlap with selection signals previously reported for Aymara or Quechua (Bigham et al. 2009, 2010). This could be the result of differences in the genotyping platform used, the statistics employed, or the choice of control population. Alternatively, our results may confirm different selection patterns among Andeans. We found only one gene candidate gene overlapping (EPO) with genes highlighted Tibetans (Bigham et al. 2010) and no common candidate gene in Ethiopians and Calchaquíes (IA).

The argument that hypoxia adaptation at IA is at least partly facilitated by genetic changes clearly needs supportive evidence from further studies. The finding of hypoxia related genes, such as *PRKG1*, among our topmost ranking genes of the selection tests suggests that candidate genes of HA adaptation may be found in IA populations. However, these preliminary results need to be followed up firstly, by sequencing studies to determine the likely causative mutations and subsequently by functional characterization of these polymorphisms. The approaches we have used here, while able to identify outlier regions of the genomes by genetic differentiation or extent of homozygosity, are to be considered with caution as the false positive rates can be high in populations with small effective population sizes (Charlesworth 2009). This is particularly due to genetic drift in isolated populations. However, Calchaquíes showed the highest heterozygosity levels among Native Americans and thus genetic drift may be a lesser concern in this population. Another limitation is the risk of confounding results due to the SNP ascertainment bias typical of genotyping methodologies (Clark et al. 2005). Whole genome sequencing should overcome this limitation. In addition, polygenic traits and alleles with slowly changing frequencies may also contribute to the genomic signatures (Pritchard et al. 2010) and are unlikely to be identified by the selection tests employed here.

Despite these limitations, our analyses identified *PRKG1* and two pathways associated with NO metabolism (Tables4 and 5) as strong follow-up candidates. Interestingly, Bigham et al. (2009, 2010) identified the inducible NO synthase as a target of selection in Aymara and Quechua. Notably, *PRKG1* was recently identified as a target of selection in Siberians (Cardona et al. 2014). The same region highlighted by PBS in this study was highlighted by XP-EHH in the North East Siberians (Chukchi, Eskimo, and Koryaks). However, high-frequency Calchaquí alleles showed only frequencies around 50% or lower in the three Siberian populations and therefore no strong case for introgression can be made. While NO is implicated in various physiological mechanisms, such as cold-induced smooth muscle constriction in Siberians, the mild climate of the Calchaquí valleys and the moderate level of hypoxia support a hypoxia-driven signal in Calchaquíes (IA). In addition, four pathways associated with the neurotransmitter GABA were significantly enriched among the top 1% XP-EHH results (Table5). GABA is increased during hypoxia (Wood et al. 1968) and protects neurons against ischemic damage (Fern et al. 1995), thus selection on GABA pathways may result in protection for the hypoxic brain.

While the overall findings of selection tests revealed only one strong candidate gene, some indicators of reduced selection pressure compared to HA Collas were observed in Calchaquíes (IA). However, we also observed shared selection signals between Collas (HA) and Calchaquíes (IA), either due to the common pressure of hypoxia or to their shared demographic history (Fig.3). To elucidate the causal factor, further comparisons of neighboring populations would be necessary.

In summary, our study suggests that developmental plasticity and/or genetic changes develop in response to even moderate hypoxia, despite the relatively recent ancestry and thus short-term exposure (in evolutionary terms) of Calchaquíes to their environment. Hence, populations living at IA (2000–3000 m) should also be considered when exploring human adaptability to hypobaric hypoxia to further elucidate influences of this selection pressure.
